# Potential for native hydrocarbon-degrading bacteria to remediate highly weathered oil-polluted soils in Qatar through self-purification and bioaugmentation in biopiles

**DOI:** 10.1016/j.btre.2020.e00543

**Published:** 2020-10-12

**Authors:** Nasser AlKaabi, Mohammad A. Al-Ghouti, Samir Jaoua, Nabil Zouari

**Affiliations:** Department of Biological and Environmental Sciences, College of Arts and Sciences, Qatar University, PoB 2713, Doha, Qatar

**Keywords:** Weathered hydrocarbons, Bioremediation, Biostimulation, Bioaugmentation, *Bacillus licheniformis*, *Pseudomonas aeruginosa*

## Abstract

Petroleum-derived contamination events constitute a unique environmental issue in the arid areas because of the weathering processes, accentuated due to harsh conditions and representing the main origin of failure of bioremediation applications. The industrial area of Dukhan and the AlZubara coast represent wethered oily-sites as an appropriate model due to the extreme weather, which characterizes Qatar. Indigenous bacteria would be highly adapted and when re-introduced or stimulated would conduct to the remediation of these sites. An appropriate approach to bioremediating-weathered oil was investigated in these two areas. Systematic soil sampling was performed, and the soil samples were analyzed. The results clearly showed the harsh chemical compositions (high salinity and contents of total petroleum components contents in the range of the oil organics). By implementation of a procedure of isolation of highly adapted bacteria, few hydrocarbon-degrading bacteria were isolated in the objective of selecting those for further application. Their identification by ribotyping showed dominance of *Bacillus* and *Virgibacillus* in AlZubara site, and *Bacillus* and *Pseudomonas* in Dukhan site. All bacterial strains were highly tolerant to 10 % diesel and showed potential of removal of 20 %–85 % of C*_10_* to C*_36_* hydrocarbons. This potential was confirmed by determination of the *n-*heptadecane/pristane and *n-*octadecane/phytane ratios, indicating in turn their potential to bioremediate oil. This was demonstrated by comparison of the self-remediation to bioaugmentation using two selected *Bacillus* and *Pseudomonas* strains using Dukhan soil in biopiles. These strains when biostimulted in such a soil increased the removal of total petroleum components to 53 % compared to 30 % with self-purification, after 90 d. The results indicated that highly adapted endogenous bacteria might be used to bioremediate highly weathered oil-contaminated soil under harsh conditions.

## Highlights

• Highly adapted hydrocarbon-degrading bacteria were isolated from weathered soils.
• High diversity of bacterial metabolism was shown although from the same soil.
• Biostimulation improves removal of weathered hydrocarbons by indigenous bacteria.
• Combination of stimulation to augmentation requires selection of indigenous strain.
• Without selection of strains, negative effect may be registered on bioremediation.

## 1 Introduction

Crude oil contains thousands of different molecules with various properties. Qatar, an important producer of oil and gas, is in an arid area with harsh soils and weather. Oil is discharged to the environment as waste during anthropogenic activities and through spillages, and oil in the environment is subjected to weathering processes, particularly under extreme conditions especially in the summer season (mean temperature 34.9 °C, relative humidity of 58 %, daily sunshine of 10.6 h, solar radiation of 584.7 mW h/sq.cm and wind speed of 26 km/h) [1,2]. In a recent study it was found that oil pollution has occurred in limited specific areas in Qatar but extensive weathering caused by the harsh weather in Qatar has led to oil in these areas being at different stages of oxidation [3]. Microbial remediation is an attractive strategy for decreasing the effects of high degrees of pollution with mixtures of complex molecules [4,5]. Soil bacteria can adapt and develop mechanisms for totally or partly degrading oil molecules to generate energy and allow the bacteria to grow. Other bacteria can use oil molecules that have already been transformed in some way [[5], [6], [7]]. Hydrocarbon-degrading bacteria can develop chemotaxis, a signaling system, to help guide access to hydrocarbons [8]. Degradation of organic compounds in oil requires bacterial cells to interact with the oil [[9], [10], [11]]. Some bacteria that have adapted to oil-contaminated soil can transfer pollutants through their hydrophobic surfaces [7,12]. Some hydrocarbon-degrading bacteria produce surfactants to help increase the solubility and accessibility of the hydrocarbons [13,14]. The susceptibility of a hydrocarbon to microbial degradation depends on the hydrocarbon structure, and branched-chain alkanes, cyclic alkanes, linear alkanes, and small aromatic compounds have different susceptibilities to microbial degradation [15,16]. Weathering processes are particularly severe in arid regions such as the area around the Persian Gulf. Biodegradation of weathered compounds is a more complex process than biodegradation of unweathered compounds [[17], [18], [19], [20]]. Only microorganisms with structures and functions well-adapted to the conditions can grow in harsh environments like the environment in Qatar. Such microorganisms are interesting because they have particular abilities to degrade or transform unconventional mixtures of organic compounds, as was found in studies performed by Al Disi et al. [19] and Attar et al. [21]. Natural microbial remediation in Qatar is therefore an appropriate model for the degradation of weathered hydrocarbons in harsh soils. It has been found in previous studies that methods for bioremediating hydrocarbons should be designed taking into account the diversity and metabolic adaptations of endogenous bacteria, particularly in areas with harsh conditions [19,22]. It has been found that a long adaptation period with endogenous bacteria is required if unadapted bacteria are used in bioaugmentation processes to remediate weathered oil-contaminated soil [23,24]. However, the fate of weathered oil in soil in arid areas like Qatar is not well investigated [25]. *In situ* microbial bioremediation by endogenous bacteria involving augmentation/stimulation processes is particularly poorly understood. Most bioremediation failures are caused by the bacteria not being adapted to harsh weather and soil [25]. Introducing exogenous bacteria cannot lead automatically to the success of the bioremediation of the soil. Interactions between the endogenous and the exogenous bacteria as well as their symbiosis in term of cooperation or inhibition are complex to predict in the case of weathered oil. Indeed, the complexity of such oil components requires, in general, potent bacteria able to tolerate their toxicity and cooperate in their removal by commensalism and/or co-metabolism. Not any of the endogenous bacteria can support the self-purification or may be seeded to accelerate the remediation of the wethered oily-soil. In order to demonstrate such hypothesis, wethered oil pollution of soil was investigated at two sites in Qatar. The study sites were in areas with unique conditions, characterized with dry soil, harsh weather conditions and weathered oil left for self-purification for more than three years [3]. They are the coastal area in AlZubara beach and the oil-waste dumping site in the industrial area of Dukhan [3]. They are considered an appropriate and a characterized model for this type of study. Here, to achieve the goal, communities representing only the highly adapted bacterial strains, tolerating high toxicity and exhibiting high activity of oil range organics, pristine and phytane were isolated, identified and screened to select candidates for seeding the soils in biopiles. Comparison between enhanced self-purification and bioaugmentation was performed, in order to establish the appropriate approach of the microbial bioremediation of weathered oily soils. The study linked the microbial, ecological and applied aspects of the issue related to the weathered oil bioremediation.

## 2 Materials and methods

### 2.1 Sampling soil polluted with weathered oil

**Fig. 1:**
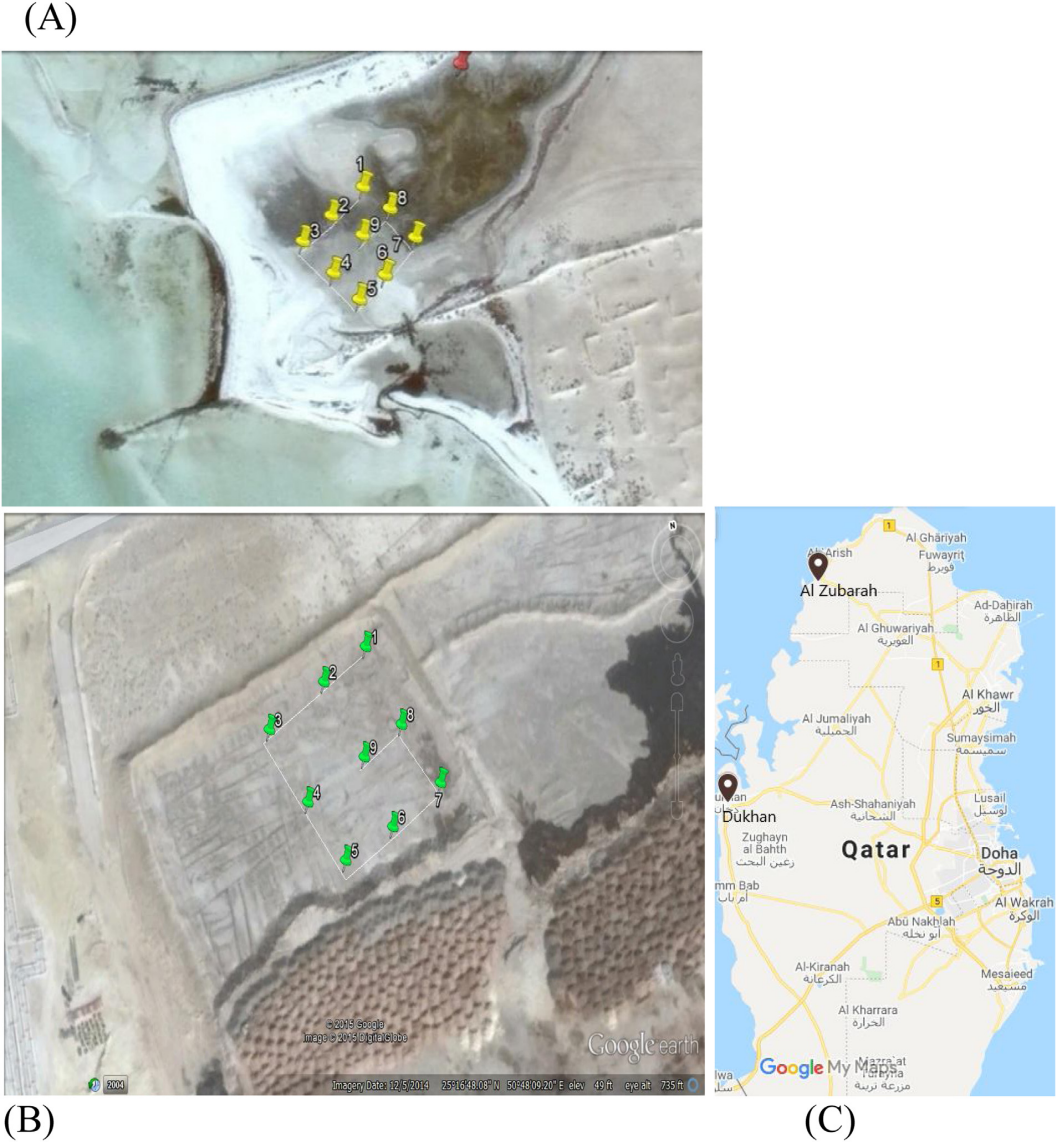
Sampling points at the (A) AlZubara beach sampling site, (B) Dukhan dump site and (C) Qatar map with the indication of the location of the 2 sites.

AlZubara beach is 12 km long and is in north-west Qatar. The beach has continually received oil pollution caused by oil transportation and related activities in the Arabian Gulf for a long time. A total of 13 sampling points along the beach were selected, and three samples of soil were collected at each of the 13 sampling points, meaning 39 samples were collected. The hydrocarbon contents and weathering statuses of all of the sampling sites were investigated in a previous study [3]. The sampling points at the AlZubara beach site are shown in Fig. 1. The Dukhan dump area is a well-controlled site that is used to store oil waste. Storing oil waste in the area has not been negatively affecting by the environment. Soil samples collected over a three-year period in which the soil was exposed to air and weathering processes have been analyzed previously [3].

Samples were collected systematically at the AlZubara and Dukhan sites. Samples were collected from nine sampling points in a square area. Each sampling point was 20 m from the neighboring sampling points. At each sampling point, a surface soil sample and a sample from a depth of 20 cm were collected, meaning a total of 36 samples were collected from both sites (18 from each site). Each soil sample was collected using a sterile spatula and was stored in a sterilized glass bottle. Each bottle was sealed, labeled, and wrapped in foil to protect the sample from light and prevent further reactions occurring. Each bottle was sealed, labeled, wrapped in foil to protect the sample from light and prevent further oxidation reactions and stored at 2−4 °C. The samples were collected in spring, and the ambient soil temperatures were 25–26 °C.

### 2.2 Preparation of soil samples for analysis

A 50 *g* aliquot of a soil sample was mixed with 50 mL of water in a 250 mL stoppered conical flask, and the mixture was shaken using a shaking device for 30 min at 25 °C. The sample was then allowed to stand for 30 min and then passed through a Whatman no. 42 filter paper (GE Healthcare Bio-Sciences, Pittsburgh, PA, USA). The filtrate was then centrifuged at 3000 rpm for 5 min.

### 2.3 Determining the major element concentrations by ion chromatography

The concentrations of various ions in the sample extracts were determined by ion chromatography using an Dionex IC 5000 system (Thermo Fisher Scientific, Waltham, MA, USA). The concentrations of chloride and sulfate (anions) and calcium, magnesium, sodium, and potassium (cations) were determined. The ion chromatography system had an isocratic pump, an injection valve with a 250 μL sample loop, a Dionex conductivity detector, and an automated sampler. A specific pH gradient was used for each analyte ion. The anions were separated using a Dionex Ionpac AS4A-SC analytical column (250 mm long, 4 mm inner diameter; Thermo Fisher Scientific) and a Dionex AG4A-SC guard column (50 mm long, 4 mm inner diameter; Thermo Fisher Scientific), and an Anion Self-Regenerating Suppressor-1 system was used. The eluent contained 1.8 mM Na_2_CO_3_ and 0.8 mM NaHCO_3_, and the flow rate was 0.25 mL/min. The cations were separated using a Dionex Ionpac CS12 analytical column (250 mm long, 4 mm inner diameter; Thermo Fisher Scientific) and a Dionex CG12 guard column (50 mm long, 4 mm inner diameter; Thermo Fisher Scientific), and a Cation Self Regenerating Suppressor system was used. The eluent contained 0.020 M methanesulfonic acid, and the flow rate was 0.25 mL/min.

### 2.4 Oil hydrocarbon analysis by gas chromatography mass spectrometry

Total Petroleum Hydrocarbons-Oil Range Organics (TPH-ORO: *n*-C10–*n*-C35) in the soils were analyzed by gas mass spectrometry according to AlKaabi et al. [3]. Accordingly, TPH were first extracted from homogenized soils, subjected to analysis, in the ASE DINOX SE 500 evaporator for extraction, using Methylene chloride/acetone (1:1, v/v) as reported [3]. The TP-ORO analysis of the extracts was performed by first dissolving an aliquot in dichloromethane to give an extract concentration of 2500 μg/mL. The solution was then analyzed using an Agilent 7890A/5975C gas chromatograph mass spectrometer (Agilent Technologies, Santa Clara, CA, USA) equipped with a 60 m ZB-5 capillary column. The carrier gas was helium, and the flow rate was 1.1 mL/min. A 1 μL aliquot of the oil solution was injected in pulsated splitless mode, and the injector temperature was 315 °C. The oven temperature program started at 40 °C, which was held for 2 min, increased at 25 °C/min to 100 °C, then increased at 5 °C/min to 315 °C, which was held for 13.4 min. The mass spectrometer was operated in selected ion monitoring mode, and *m/z* 55 was monitored [26].

### 2.5 Analysis of hydrocarbons in diesel by gas chromatography flame ionization detection

Hydrocarbons in diesel were determined using an Agilent 6890 N gas chromatograph with a flame ionization detector (Agilent Technologies). This type of analysis is usually performed to investigate diesel degradation. Separation was achieved using an Agilent HP-1 gas chromatography column (30 m long, 0.25 mm inner diameter, 0.10 μm film thickness, on a 7-inch cage; Agilent Technologies). The oven temperature program started at 100 °C, then increased at 15 °C/min to 280 °C, which was held for 5 min. The carrier gas was nitrogen, and the pressure was 7.85 psi, giving a starting flow rate of 6.1 mL/min. Once a chromatogram had been acquired, the concentration of each analyte was determined from the peak area using US Environmental Protection Agency method #8015.

### 2.6 Enrichment of cultures for isolating hydrocarbon-degrading bacteria

Cultures containing hydrocarbon-degrading bacteria were prepared from samples of surface soil (the upper soil layers) and soil from 20 cm deep (the lower soil layers) from each sampling point and samples of oil waste. A 1 g aliquot of a sample was suspended in 20 mL of Luria Broth (LB). The mixture was then incubated at 30 °C for 72 h on a shaker set to shake at 300 rpm. A 2 mL aliquot of the liquid was then added to 20 mL of mineral salt medium (MSM) supplemented with 1 mL of diesel or crude oil as a carbon source. This adaptation step was repeated three times to ensure that the medium was enriched with bacteria capable of growing using hydrocarbons in crude oil or diesel [19,21,22]. A 100 μL aliquot of an enriched LB liquid culture was then spread on MSM agar, then 100 μL of crude oil or diesel was sprayed onto the agar. Isolates with distinct morphologies were transferred to new LB agar plates and purified by successively sub-culturing the isolated colonies six times. The MSM contained 4.0 g/L NH_4_NO_3_, 2.0 g/L Na_2_HPO_4_, 0.53 g/L KH_2_PO_4_, 0.17 g/L K_2_SO_4_, 0.10 g/L MgSO_4_.7H_2_O, 1 g/L ethylenediaminetetraacetic acid, 0.42 g/L ZnSO_4_, 1.78 g/L MnSO_4_, 0.5 g/L H_3_BO_3_, and 1 g/L NiCl_2_. MSM solid medium was prepared by adding 20 g of agar to 1 L of MSM.

### 2.7 Molecular identification of the hydrocarbon-degrading bacterial isolates

The DNA from cells grown overnight on a LB plates was removed using a polymerase chain reaction (PCR) protocol. The cells were suspended in 0.2 mL of distilled water and stored at −80 °C for 20 min. The cells were then placed in a water bath at 100 °C for 10 min. The mixture was then centrifuged for 10 min at 13,000 rpm, and the supernatant was transferred to a new tube and subjected to the next part of the PCR protocol. The 16S rDNA was amplified using the universal PCR primers RibS73sp (AGAGTTTGATCCTGGCTCAG) and RibS74sp (AAGGAGGTGATCCAGCCGCA) [27]. The PCR protocol was performed using 25 μL of PCR buffer containing 1.5 μM MgCl_2_, 0.8 μM deoxyribonucleotide triphosphate, 1.35 μM forward and reverse primers, 10–20 ng of isolated genomic DNA, and 0.5 IU of Taq DNA. The PCR reaction started with denaturation at 94 °C for 3 min, then had 35 cycles of 45 s denaturation at 94 °C, 45 s annealing at 50 °C, and 45 s elongation at 72 °C, and then had a final 2 min extension step at 72 °C. DNA purification was performed using a QIAquick gel extraction kit. Bacterial 16S rRNA amplicons were sequenced after the amplicons had been purified. Sequencing data were generated by the sequencing unit. Sequences lengths were between 900 bp and 970 bp. The 16S rDNA sequence for each isolate was used to identify related DNA sequences in the Gene Bank database using the Blast server at the US National Center for Biotechnology Information.

### 2.8 Self-purification and bioaugmentation in soil biopiles

`The method used to investigate self-purification and bioaugmentation was previously published by Oualha et al. [28]. Biopiles were performed in glass containers (20 × 10 × 15) cm containing 685 g soil each. The C/N/P ratios mentioned with results were adjusted using ammonium nitrate containing 34 % (w/w) nitrogen and potassium phosphate containing 22 % (w/w) phosphorous as reported by Oualha et al. [28]. Water content of all biopiles was adjusted to the indicated level, using dilled water [28]. The biopiles were covered with aluminium foil preventing light oxydation of hydrocarbons and incubated at in an incubator set at the desired temperature. All biopiles were mixed, manually, twice a week during the period of incubation. For biopiles performed with seeding, a 5 mL suspension of the pellet of the selected isolates was prepared from a 4-day culture in 20 mL-liquid MSM medium supplemented with 10 % diesel and washed twice with MSM. The suspension was then mixed in the corresponding biopile-soil. The bacterial cells in the solution and soils were then counted and the number of colony-forming units (CFU) was determined.

### 2.9 Determining bacterial cell counts in the liquid and soil samples

The bacterial cell count for a sample was determined by performing serial dilutions and then spreading a diluted sample on LB medium and incubating the plate at 30 °C for 48 h. For a liquid, a 1 mL aliquot was serially diluted and then, for each dilution, 100 μL of the solution was spread on a solid LB plate. The dilution factor was considered when calculating the cell densities. For soil, 1 g of soil was suspended in 1 mL of liquid MSM, then the mixture was serially diluted with MSM and treated as described above for a liquid sample.

### 2.10 Statistical analysis

Each experiment was performed in triplicate and the mean result was calculated. Each mean is presented below with the standard deviation. The means and standard deviations were calculated using Microsoft Excel 2013. The significances of differences between sets of results were determined by performing one-way analyses of variance using the 95 % confidence level (p > 0.05).

## 3 Results and discussion

### 3.1 Chemical and physical characterization of the AlZubara and Dukhan soil samples

The samples from AlZubara beach were collected from a site that was found to contain weathered oil in a previous study [3]. Soil from Dukhan that had been weathered for 3 years was used as a source of adapted bacteria. AlZubara beach was continuously under pollution by Oil as exposed to oil industry in the Gulf sea. AlKaabi et al. [3] showed that such pollution is continuous and traces of petroleum dated from the Gulfa War in 1991. A composite sample from each site (AlZubara and Dukhan) was prepared, by mixing 5 kg of soil sample from each site, meaning that 45 kg from all the surfaces and 45 kg from 20 depth were homogenously prepared. These samples served to analyze the major composition in each site.

The chemical and physical characteristics of the samples from AlZubara and Dukhan were determined to allow the environmental conditions suitable for the indigenous microbial communities found at the sites to be evaluated, and the results are shown in Table 1.

**Table 1:** Chemical composition and physical characteristics of the soil sampled from the upper and lower layers from AlZubara and Dukhan sites. TPH: Total Petroleum Hydrocarbons, DRO: Diesel Range Organics, ORO: Oil Range Organics. The values were calculated statistically and are the average of three separate determinations.

Component	AlZubara site		Dukhan site	
	Surface layer	20 cm deep	Surface	20 cm deep
Na (mg/g)	11 ± 1	11 ± 1	1.9 ± 0.8	1.9 ± 0.8
K (mg/g)	0.40 ± 0.01	0.40 ± 0.01	0.09 ± 0.05	0.10 ± 0.04
Mg (mg/g)	0.99 ± 0.05	0.99 ± 0.06	0.114 ± 0.005	0.113 ± 0.006
Ca (mg/g)	0.44 ± 0.02	0.44 ± 0.02	1.143 ± 0.007	1.148 ± 0.008
Cl (mg/g)	19.4 ± 0.9	19 ± 1	2.4 ± 0.2	2.4 ± 0.1
Br (mg/g)	0.082 ± 0.003	0.082 ± 0.003	0.057 ± 0.005	0.056 ± 0.004
NO_3_ (μg/g)	0.007 ± 0.001	0.0078 ± 0.0009	0.002 ± 0.004	0.0023 ± 0.0005
SO_4_ (mg/g)	2.04 ± 0.06	2.04 ± 0.05	0.18 ± 0.01	0.17 ± 0.02
NO_2_ (μg/g)	0.136 ± 0.008	0.135 ± 0.007	0.025 ± 0.003	0.023 ± 0.002
PO_4_ (μg/g)	0.35 ± 0.03	0.36 ± 0.03	0.09 ± 0.01	0.08 ± 0.01
Salinity (ppt)	5.9 ± 0.1	6.0 ± 0.1	2.39 ± 0.07	2.38 ± 0.04
pH	7.20 ± 0.06	7.21 ± 0.08	6.73 ± 0.06	6.75 ± 0.06
TPH-DRO (μg/kg)	<1.0	<1.0	6250 ± 290	6480 ± 410
TPH-ORO (mg/kg)	280 ± 20	275 ± 16	39,700 ± 1300	40,600 ± 1400

The overall chemical and physical characteristics of the surface layer and 20 cm below the surface of the soil from the AlZubara or Dukhan sites were not significantly different (one-way analysis of variance, p > 0.05). The total petroleum hydrocarbon (TPH) oil range organic (ORO) contents of the upper and lower soil samples were around 280 mg/kg, and the TPH diesel range organic (DRO) contents were <1 μg/kg. The upper and lower soil samples were all slightly alkaline (∼pH 7.20). The sulfate contents of the upper and lower soil samples were around 2.04 mg/g, the nitrate and ammonia contents were low (∼0.140 μg/g and ∼0.007 μg/g, respectively), and the phosphate contents were moderate (∼0.350 μg/g). There were also no significant differences between the characteristics of the upper and lower soil samples from the Dukhan site. However, most of the mineral contents were significantly lower in the Dukhan soil samples than the AlZubara soil samples, but the calcium contents were twice as high in the Dukhan soil samples as in the AlZubara soil samples. The salinities were low in the samples from both sites. They are of 2.4 ppt and 6 ppt in Dukhan and AlZubara sites, respectively, indicating that the samples may have not contained halophilic and halotolerant microorganisms [29]. The Dukhan soil samples were slightly acidic (pH 6.75). The pH values of the soil samples from both sites were likely to be favorable for most microorganisms that could degrade hydrocarbons. The TPH-DRO contents of the upper and lower Dukhan soil samples were 6250 and 6480 mg/kg, respectively. The TPH-ORO contents of the upper and lower Dukhan soil samples were ∼4000 mg/kg. This indicated that soil at the Dukhan site was much more polluted with TPH than was soil at the AlZubara site. This could be because solid and liquid oil waste from oil extraction activities are collected at specific sites in the Dukhan area. When a specific dump area is full it is left open to the air for years to allow self- remediation to occur. The Dukhan site is strictly controlled to prevent spreading of oil pollution or the transfer of oil to groundwater or watercourses. However, the site is subjected to weathering processes.

### 3.2 Isolation of potential hydrocarbon-degrading bacteria from AlZubara and Dukhan

Eight bacterial isolates were prepared from the upper and lower soil samples from the nine sampling points in the AlZubara area. Because of the isolation procedure that was used, the isolates were expected to contain hydrocarbon-degrading bacteria. The isolates were identified by ribotyping based on 16S rDNA sequencing, and similarities between the sequences and sequences in the Blast database were identified. The results are shown in Table 2.

**Table 2:** Bacteria isolated from the AlZubara soil samples. The sampling point numbers are the numbers shown in Fig. 1. Each code is for one purified species/strain. The similarities and accession numbers were obtained after the DNA sequences had been deposited in the Gene Bank database using the Blast server at the US National Center for Biotechnology Information.

Sampling point	Layer	Code	Identification	Similarity	Accession number
Point 3	Surface	Z3S1	*Bacillus subtilis*	100 %	AF549498.1
	Lower	None	–	–	–
Point 4	Surface	None	–	–	–
	Lower	Z4D1	*Bacillus licheniformis*	100 %	LN995452.1
Point 6	Surface	Z6S1	*Providencia rettgeri*	99 %	CP027418.1
	Lower	Z6D1	*Virgibacillus marismortui*	99 %	MF321845.1
Point 7	Surface	Z7S1	*Providencia rettgeri*	99 %	CP027418.1
	Lower	Z7D1	*Virgibacillus halodenitrificans*	99 %	KT945027.1
Point 8	Surface	None	–	–	–
	Lower	Z8D1	*Morganella morganii*	98 %	KU942503.1
Point 9	Surface	None	–	–	–
	Lower	Z9D1	*Bacillus circulans*	100 %	KY849415.1

It can be seen that few isolates were isolated. There are two possible reasons for this. First, the soil samples had low contents of organic compounds required for cell growth and maintenance. Therefore, few cells would have been able to adapt and subsist. Second, the aim of the isolation strategy was to isolate hydrocarbon-degrading bacteria by enriching cultures in MSM containing 10 % diesel. This culture medium was very toxic to bacteria because the hydrocarbon compound concentration was 75 g/L (the total hydrocarbon concentration in the diesel was 750 g/L, and the diesel concentration in the culture medium was 10 % v/v). The aim of the isolation procedure was to isolate and purify hydrocarbon-degrading bacteria with strong potentials to degrade and tolerate diesel and crude oil. The isolation procedure was previously published by Al Disi et al. [19] who demonstrated such a process of isolated of highly potent hydrocarbon-degrading bacteria. The isolated bacteria were therefore highly adapted. However, it was clear that these bacterial isolates were not homogeneously distributed in all of the samples and both layers. The samples from some sampling points (numbers 1, 2, and 5) did not contain any adapted isolates. For other sampling points, only the upper or lower samples contained adapted bacterial isolates. Of the eight isolates, three bacterial strains belonged to the *Bacillus* genus but were of three different species (*Bacillus subtilis*, *Bacillus licheniformis*, and *Bacillus circulans*). Two strains of *Providencia rettgeri* were isolated. Both strains were found in surface soil samples. Two isolates belonged to the *Virgibacillus* genus. One was the species *Virgibacillus halodenitrificans* and the other was the species *Virgibacillus marismortui*. Both *Virgibacillus* species were found only in lower soil samples. One *Morganella morganii* strain was isolated from the samples from the AlZubara site. *Bacillus licheniformis* has previously been found to degrade hydrocarbons [19]. Some strains of *Bacillus subtilis* also degrade hydrocarbons [29]. Three strains of *Providencia rettgeri* have been found within oil bacterial populations, and responsible of the degradation of oil organic compounds [30]. Some *Bacillus* strains have previously been isolated from soil from polluted sites in Qatar and were found to degrade hydrocarbons [19,28]. The other genera of bacteria have not previously been found in the environment in Qatar or other areas around the Arabian Gulf. A total of 16 isolates were obtained from the upper and lower soil samples from the nine sampling points at the Dukhan site. The isolates are described in Table 3, and the sampling point each isolate was found at and the identity of each isolate determined by ribotyping (based on 16S rDNA sequencing) are also shown. Like for the AlZubara site, few isolates were found at the Dukhan site because the aim of the isolation procedure was to isolate hydrocarbon-degrading bacteria with strong potentials to degrade and tolerate diesel and crude oil.

**Table 3:** Bacterial strains isolated from the Dukhan soil samples. The point numbers are the numbers of the systematic sampling points. Each code is for one purified species/strain. The similarities and accession numbers were obtained after the DNA sequences had been deposited in the Gene Bank database using the Blast server at the US National Center for Biotechnology Information.

Sampling point	Layer	Code	Identification	Similarity	Accession number
Point 1	Surface	D1S1	*Bacillus* sp.	100 %	KY911251.1
	Lower	D1D1	*Bacillus* sp.	100 %	MG855692.1
	Lower layer	D1D2	*Bacillus licheniformis*	99 %	KY962349.1
Point 2	Surface	D2S2	*Pseudomonas luteola*	100 %	KC429633.1
	Lower	D2D2	*Bacillus subtilis*	100 %	MH071337.1
		D2D3	*Bacillus subtilis*	100 %	MH040981.1
Point 4	Surface	D4S2	*Pseudomonas luteola*	100 %	NR_114215.1
	Lower	D4D3	*Pantoea calida*	99 %	KX036541.1
Point 5	Surface	D5S1	*Pseudomonas aeruginosa*	100 %	KF261029.1
	Lower	D5D1	*Pseudomonas aeruginosa*	100 %	KY040017.1
Point 6	Surface	D6S2	*Pseudomonas luteola*	100 %	KX301304.1
	Lower	None	–	–	–
Point 7	Surface	D7S1	*Pseudomonas aeruginosa*	100 %	KY040014.1
	Lower	D7D1	*Bacillus sp.*	100 %	MG835309.1
Point 8	Surface	None	–	–	–
	Lower	D8D2	*Pseudomonas stutzeri*	99 %	MF421776.1
Point 9	Surface	D9S1	*Pseudomonas stutzeri*	99 %	KX180912.1
	Lower	D9D1	*Pseudomonas aeruginosa*	100 %	KF598858.1

In terms of biodiversity at the Dukhan site, the genus *Pseudomonas* was represented by nine strains of three species. The *Bacillus* genus was represented by six strains of several different species isolated from both the upper and lower soil samples. These *Bacillus* and *Pseudomonas* species were found in both upper and lower soil samples from different sampling points. *Pantoea calida* was found only in the lower soil sample from sampling point 4. Some *Bacillus* species, *e.g.*, the subspecies *Bacillus lichenoformis* and *Bacillus subtilis* [31,32] have previously been found to degrade hydrocarbons. *Pseudomonas luteola* has previously been described [33]. *Pseudomonas aeruginosa* strongly degrades hydrocarbons [34] as does *Pseudomonas stutzeri* [35]. *Pantoea calida* has not previously been found to degrade hydrocarbons. Some of the hydrocarbon-degrading *Bacillus* and *Pseudomonas* bacteria isolated from the Dukhan soil samples may have been very similar strains because only a short sequence of 16S rDNA was amplified when the ribotyping identification procedure was performed.

### 3.3 Potentials for the isolated bacteria to degrade diesel hydrocarbons

Differences between the isolated strains were determined from the different biological activities of the strains in MSM containing 10 % v/v diesel (the only source of carbon). The diesel concentration of 10 % corresponded to a hydrocarbon concentration of 75 g/L (the hydrocarbon concentration in the diesel was 750 g/L). The potentials of each strain to grow using hydrocarbons as an energy source and to tolerate high hydrocarbon concentrations were also investigated. The potential of each strain to adapt to the presence of hydrocarbons by synthesizing biosurfactants to increase hydrocarbon bioavailability, by removing hydrocarbons with low molecular weights (LMWs), medium molecular weights (MMWs), and high molecular weights (HMWs), and by removing TPH was also investigated. The *n*-heptadecane (*n-*C17) to pristane ratio and *n*-octadecane (*n-*C18) to phytane ratio for the medium containing each strain were determined to allow the potential for that strain to biodegrade oil to be assessed. The results for all of the strains isolated from the soil samples from the AlZubara and Dukhan sites are shown in Table 4. The results indicated that all of the strains isolated from the soil samples from the AlZubara site were able to grow and increase the cell biomass under the experimental conditions that were used. However, different amounts of biomass were produced by the different strains. The TPH removal efficiencies were clearly not proportional to the amounts of biomass produced. For example, strain Z3S1 gave a final CFU of 0.15 × 10^7^ per mL and removed 38 % of the TPH, but Z4D1 gave a final CFU of 1.33 × 10^7^ per mL and removed 19 % of the TPH (half of the percentage removed by Z3S1). These results reflected the different metabolic pathways used by the different bacteria caused by metabolic diversity and the adaptation processes the bacteria used. The TPH removal efficiency was used as a criterion to differentiate between the different isolates. Five strains (the largest group of strains) removed 19 %–23 % of the TPH, and two strains (the second largest group) removed 27 %–29 % of the TPH. Interestingly, Z3S1 removed 38 % of the TPH, which was a higher percentage than was removed by any other isolate. Most of the isolates removed 13 %–18 % of the LMW (*n-C*12–*n-C*16) hydrocarbons, but Z7D1 removed 27 % of the LMW hydrocarbons. Z7D1 also removed 29 % of the HMW (*n-C*21–*n-C*25) hydrocarbons, which was a higher percentage than was removed by any other isolate. There were also differences between the percentages of the MMW (*n-C*17–*n-C*20) hydrocarbons by the different isolates. Z3S1 gave the highest MMW and HMW hydrocarbon removal efficiencies, of almost 35 %.

**Table 4:** Screening results for the bacterial strains isolated from the soil samples from the AlZubara and Dukhan sites by culturing the bacteria in mineral salt medium containing 10 % diesel (SA: solubilization activity, TPH: Total Petroleum Hydrocarbons, EA: emulsification activity, LMW: low molecular weight (*n-C*12–*n-C*16) hydrocarbons, MMW: medium molecular weight (*n-C*17–*n-C*20) hydrocarbons, HMW: high molecular weight (*n-C*21–*n-C*25) hydrocarbons). The values were calculated statistically and are the average of three separate determinations. The control is the non-inoculated culture.

Strain	Growth (10^7^ CFU/mL)	EA (U/mL)	SA (%)	TPH removal(%)	LMW removal (%)	MMW removal (%)	HMW removal (%)	*n-*C17 /pristane ratio	*n-*C18/ phytane ratio
Z3S1	0.15 ± 0.01	9.9 ± 0.3	0.16 ± 0.04	38.0 ± 0.6	14.9 ± 0.9	35.2 ± 0.8	33.9 ± 0.3	4.3	8.0
Z4D1	1.33 ± 0.01	1,7 ± 0.4	2.33 ± 0.03	19.0 ± 0.7	10.0 ± 0.8	18.2 ± 0.4	24.5 ± 0.7	9.3	5.7
Z6S1	0.10 ± 0.02	2.6 ± 0.6	0.5 ± 0.1	22.0 ± 0.5	18.1 ± 0.8	26.9 ± 0.7	22.3 ± 0.8	6.6	2.4
Z6D1	0.05 ± 0.01	6.4 ± 0.8	1.10 ± 0.05	22.0 ± 0.6	17.2 ± 0.6	21.7 ± 0.8	21.2 ± 0.6	6.0	5.3
Z7S1	0.06 ± 0.01	2.8 ± 0.2	1.90 ± 0.05	23 ± 0.6	27.5 ± 0.9	18.2 ± 0.6	28.7 ± 0.7	7.2	4.7
Z7D1	0.16 ± 0.02	1.1 ± 0.6	6.31 ± 0.05	29 ± 1.2	17.6 ± 0.5	22.8 ± 0.5	24.5 ± 0.9	2.4	10.4
Z8D1	0.09 ± 0.01	2.3 ± 0.6	1.03 ± 0.04	28 ± 0.9	16.9 ± 0.9	12.8 ± 0.7	15.2 ± 0.7	15	2.5
Z9D1	0.08 ± 0.01	5.2 ± 0.7	0.20 ± 0.01	23.0 ± 0.9	13.2 ± 0.7	24.0 ± 0.8	26.4 ± 0.5	9.0	23.0
D1S1	0.49 ± 0.04	6.5 ± 0.1	5.37 ± 0.04	28.7 ± 0.7	24.5 ± 0.8	20.6 ± 0.9	19.2 ± 0.6	3.0	2.0
D1D1	0.47 ± 0.04	8.2 ± 0.9	15.1 ± 0.4	31.1 ± 0.6	29.3 ± 0.7	29.2 ± 0.7	44.2 ± 0.7	9.5	4.4
D1D2	2.67 ± 0.03	303 ± 1	28.20 ± 0.09	48.1 ± 0.9	78.6 ± 0.8	61.7 ± 0.8	84.9 ± 0.9	58.5	65.3
D2S2	0.20 ± 0.05	16 ± 1	19.0 ± 0.5	21.3 ± 0.8	27.5 ± 0.6	31.8 ± 0.9	33.6 ± 0.6	17.4	8.1
D2D2	0.47 ± 0.04	19.2 ± 1	18.2 ± 0.5	17.6 ± 0.8	9.6 ± 0.8	24.4 ± 0.9	20.2 ± 0.7	18.4	6.5
D2D3	0.37 ± 0.04	19 ± 1	17.2 ± 0.4	16.5 ± 0.7	12.6 ± 0.7	23.2 ± 0.7	21.1 ± 0.8	19.4	4.3
D4S2	0.43 ± 0.04	16 ± 1	16.2 ± 0.5	24.5 ± 0.8	19.6 ± 0.8	33.6 ± 0.7	17.3 ± 0.9	6.2	8.5
D4D3	0.17 ± 0.04	14 ± 1	13.3 ± 0.6	17.5 ± 0.6	15.5 ± 0.7	16.3 ± 0.9	19.0 ± 0.7	7.1	15.5
D5S1	13.7 ± 0.1	113 ± 1	107.0 ± 0.9	23.4 ± 0.7	7.9 ± 0.6	19.3 ± 0.7	19.9 ± 0.8	10.1	8.7
D5D1	14.7 ± 0.1	279 ± 9	238.0 ± 1.1	42 ± 1	24.6 ± 0.7	38.3 ± 0.8	43.6 ± 0.7	38.3	31.7
D6S2	0.10 ± 0.02	8.5 ± 0.9	12.4 ± 0.6	26.5 ± 0.7	18.4 ± 0.5	29.1 ± 0.9	17.3 ± 0.8	3.4	9.8
D7S1	5.37 ± 0.06	116 ± 1	16.6 ± 0.5	27.0 ± 0.8	17.2 ± 0.6	19.9 ± 0.9	18.9 ± 0.7	13.2	9.0
D7D1	1.43 ± 0.08	17 ± 1	13.9 ± 0.7	17.6 ± 0.7	22.7 ± 0.8	19.3 ± 0.8	19.1 ± 0.8	16.5	14.2
D8D2	0.60 ± 0.05	8.2 ± 0.3	2.2 ± 0.3	19.2 ± 0.8	10.8 ± 0.7	18.0 ± 0.9	34.7 ± 0.9	14.3	5.4
D9S1	4.70 ± 0.09	13 ± 1	12.2 ± 0.8	14.7 ± 0.8	28.6 ± 0.7	14.1 ± 0.8	15.3**±** 0.8	5.43	3.6
D9D1	3.33 ± 0.09	178 ± 3	18.2 ± 0.8	19.2 ± 0.9	9.6 ± 0.8	19.1 ± 0.7	17.1 ± 0.8	25.2	19.5

The biosurfactant activities in the culture broths were determined to evaluate the abilities of the bacteria strains to enhance diesel biodegradation. Biosurfactants are essential to bioremediation because they emulsify and solubilize hydrophobic compounds. The biosurfactant activities in the culture broths of all of the bacteria strains were weak. As mentioned above, biosurfactant activity is essential for bacteria to interact with hydrocarbons, so these results may have been caused by the biosurfactants that were produced having been fully engaged with hydrocarbons and therefore removed with the organic phase before the analysis was performed or by the biosurfactants being attached to cell walls. Intracellular biosurfactants in natural systems tend to become attached to cell walls or excreted [36]. A bacterial cell has a membrane made up of lipids, and the transportation of insoluble substrates through the membrane is facilitated by intracellular biosurfactants that can pass through the membrane. Complex lipids, proteins, and carbohydrates are extracellular biosurfactants that facilitate the solubilization of substrates that are potentially useful to bacteria [37]. The main difference between an intracellular and extracellular biosurfactant is the chemical nature of the hydrophilic head [36]. The isolate Z3S1 had a higher emulsification activity (9.9 EU/mL, where EU is emulsification units) than the other isolates, and Z6S1 gave the next highest (6.4 EU/mL). All of the isolates had solubilization activities, however poor. It was therefore clear that the isolates had a high range of hydrocarbon-degradation activities. Some isolates were expected to have complementary activities.

The *n-*C17/pristane ratio and *n-*C18/phytane ratio were used to indicate biodegradation assuming that the isoprenoid hydrocarbons pristane (*n-*C19) and phytane (*n-*C20) had similar volatilities to *n-*C17 and *n-*C18 and that if they disappeared at different rates it would be caused by a mechanism (*e.g.*, biodegradation) other than evaporation [24]. Isoprenoids are less susceptible than *n*-alkanes of similar molecular weights to microbial degradation. The rates at which isoprenoids evaporate and are degraded tend to decrease as the degree of alkylation increases [37]. For example, the isolate Z8D1 had the highest *n-*C17/pristane ratio of 15 %, but Z4D1 and Z9D1 had *n-*C17/pristane ratios of only 9%. The other isolates had *n-*C17/pristane ratios of 2.3 %–7%. The isolate Z9D1 had the highest *n-*C18/phytane ratio of 23 %, and Z6S1 and Z7S1 had *n-*C18/phytane ratios of 15 % and 10 %, respectively, but the other isolates had *n-*C18/phytane ratios of 2.5 %–8%. All of the isolates from the soil samples from Dukhan were able to grow under the experimental conditions and produce new biomass. However, the isolates, even species within the same genus, had very different potentials for producing new cell biomass. The amounts of biomass produced were not proportional to the TPH removal efficiencies. For example, D1D1 gave a final CFU of 0.47 × 10^7^ per mL and removed 31 % of the TPH, but D1D2 gave a final CFU of 2.67 × 10^7^ per mL and removed 48 % of the TPH. These results reflected the metabolic diversity and adaptation processes of the isolates. Interestingly, D1D2 (*Bacillus licheniformis*) and D5D1 (*Pseudomonas aeruginosa*) removed 48 % and 42 % of the TPH, respectively, under the experimental conditions. The TPH removal efficiencies for most of the isolates were 16 %–28 %. Most the isolates, including D5D1, removed <30 % of the LMW (*n-C*12–*n-C*16) hydrocarbons, but D1D2 removed 72 %. Most of the isolates degraded MMW and HMW hydrocarbons moderately, giving removal efficiencies <30 %, but D1D2 degraded 60 % of the MMW hydrocarbons and 80 % of the HMW hydrocarbons and D5D1 removed 38 %–43 % of the MMW and HMW hydrocarbons. Very small amounts of biosurfactants were found in the culture supernatants. We therefore drew similar conclusions about the biosurfactants produced by the Dukhan samples as we drew for the AlZubara samples. The biosurfactants produced by the isolated bacteria would have been intracellular if they promoted the transportation of insoluble substrates through the membranes [[38], [39], [40]]. Isolate D1D2 had the highest emulsification activity (303 EU/mL), followed by the strain D5D1 (278 EU/mL) and isolates D9D1, D7S1, and D5S1 had emulsification activities of 178, 116, and 113 EU/mL, respectively. It is clear from these results that the 16 isolates from the Dukhan soil samples had very different hydrocarbon-degradation activities from the isolates from the AlZubara beach samples. The isolates tolerated hydrocarbon toxicity well and degraded hydrocarbons at high concentrations. Strong selection pressure would have led to bacteria most appropriate for bioremediating oil hydrocarbons being isolated. It would be expected that some isolates would have had complementary activities. Isolate D1D2 had the highest *n-*C17/pristane ratio (58 %), and the D5D1 *n-*C17/pristane ratio was 38.3 %. The other isolates had *n-*C17/pristane ratios of 2.4 %–25 %. D1D2 had the highest C18/pristane ratio (65 %), D5D1 had the next highest (31.68 %), and the other isolates had *n-*C18/ phytane ratios of 2.5 %–23 %. D1D2 (*Bacillus licheniformis*) and D5D1 (*Pseudomonas aeruginosa*) had the strongest abilities to biodegrade the highly weathered hydrocarbons found at the Dukhan site. The significance of differences between D1D2 and D5D1 results in one side and of all the rest of the strains on the other side, regarding the biodegradation ability (*n-*C17/pristane and *n-*C18/phytane ratios), removal of HMW range of hydrocarbons and emulsification activities was clear by performing one-way analyses of variance using the 95 % confidence level (p > 0.05). They were selected for further studies.

### 3.4 Bioremediation of weathered Dukhan soil through self-purification and bioaugmentation in biopiles

The TPH-ORO contents of the AlZubara soil samples were around 280 mg/kg, but the TPH-DRO contents were <1 μg/kg. The TPH-DRO contents of the upper and lower Dukhan samples were 6250 and 6470 mg/kg, respectively. The TPH-ORO contents of the upper and lower Dukhan samples were around 4000 mg/kg. This indicated that the Dukhan soil samples were much more polluted with TPH than the AlZubara soil samples. The self-bioremediation potential was therefore studied using the Dukhan soil samples. The bioaugmentation approach was tested using isolates D1D2 (*Bacillus licheniformis*) and D5D1 (*Pseudomonas aeruginosa*). A biopile system was used to perform *ex situ* bioremediation tests under laboratory conditions. Each biopile contained 685 g of homogeneous soil (a mixture of the upper and lower soil samples) that had been passed through a 2 mm sieve to remove particles with diameters >2 mm. The C/N/P ratio is very important for bacterial growth, so the carbon, nitrogen, and phosphorus contents of the weathered Dukhan soil samples were determined. The results are shown in Table 5.

**Table 5:** Characteristics of the oil-polluted soil from Dukhan (TPH: Total Petroleum Hydrocarbons, DRO: Diesel Range Organics, ORO: Oil Range Organics). The values were calculated statistically and are the average of three separate determinations.

Component	Content
Total nitrogen (mg/kg)	0.50 ± 0.02
Total phosphorus (mg/kg)	0.09 ± 0.03
Total carbon (mg/kg	238 ± 4
Total hydrogen (mg/kg)	20 ± 1
pH	6.75 ± 0.06
TPH-DRO (μg/kg)	6480 ± 410
TPH (ORO) (mg/kg)	40,600 ± 1400
Moisture (%)	6.48 ± 0.06

As expected, because the soil was heavily polluted, the carbon and hydrogen contents of the soil were high, and contributed around 23 % of the dry matter content. The total nitrogen and phosphorus contents of the soil were low, at 0.5 and 0.09 mg/kg respectively, which was also expected. The C/N/P ratios for the homogeneous soil were 238/0.5/0.09 (which could be expressed as 100/0.21/0.038), which was not appropriate for microbial growth in the bioremediation system. The optimal C/N/P ratios for the bioremediation of oil by bacteria are between 100/10/0.5 and 100/20/1 [41]. The C/N/P ratios were therefore adjusted to the ratios shown in Table 6 for use in the different bioremediation tests. The ratios were adjusted by adding ammonium nitrate and potassium phosphate, as mentioned in the “Materials and methods” section. The pH of the soil was close to 7. The TPH-DRO content was 6.4 mg/kg, and the TPH-ORO content was 40,270 mg/kg. The moisture contents of the soil used in the different tests were adjusted to 10 % or 13.5 % by adding distilled water. In fact, preliminary results (not shown) showed growth of all isolated bacteria in soils containing 10 % or 13.5 % moisture. Knowing that the soil initially contained 6% moisture and considering the dry weather in the region for long periods of the year, these two moisture contents were selected for the study. The biopiles were incubated at room temperature (23 °C) or at 30 °C. The growth of the endogenous bacteria (as the total CFU) and the TPH contents were periodically determined. The values found after 90 d are shown in Table 6. As expected, bioremediation of the oil-contaminated soil required the chemical composition of the soil to be adjusted to provide the optimal nutritional requirements of the hydrocarbon-degrading microorganisms [41]. Ammonium nitrate was found to be an appropriate source of nitrogen for the endogenous hydrocarbon-degrading bacteria in the Dukhan soil. Adjusting the moisture content to either 10 % or 13.5 % was necessary. Increasing the temperature from room temperature to 30 °C increased the bacterial growth efficiency and increased the percentage of TPH removed. The TPH-DRO and TPH-ORO removal efficiencies through self-purification were 30 % and 20 %, respectively, after 90 d at 30 °C at a moisture content of 13.5 %. Interestingly, the soil pH remained close to 7, meaning incubating the soil for longer could have given a better self-purification performance. Bioaugmentation using D1D2 (*Bacillus licheniformis*) and D5D1 (*Pseudomonas aeruginosa*) increased the TPH-DRO and TPH-ORO removal efficiencies to 53 % and 30 %, ORO respectively. Bioaugmentation with D1D2 and D5D1 did not appear to cause inhibition of growth of either the endogenous bacteria or the bioaugmented bacteria themselves, although there would have been very complex interactions between all of the bacteria in the soil.

**Table 6:** Bioremediation parameters for the Dukhan soil samples in the biopiles (TPH: Total Petroleum Hydrocarbons, DRO: Diesel Range Organics, ORO: Oil Range Organics). The ratios represent the C/N/P ratios in the corresponding biopile. Calculation of the removals (%) was based on the determinations performed after 90 d incubation and the initial ones. The values were calculated statistically and are the average of three separate determinations.

Pile#: C/N/P ratio (temperature)	Moisture content (%)	Final pH	Bacterial growth (10^6^ CFU/g)	TPH-DRO removal (%)	TPH-ORO removal (%)
#1: Control 100/0.23/0.02 (23 °C)	6.48	7.2	0.00213 ± 0.00006	6.34 ± 0.07	2.7 ± 0.2
#2: Control 100/0.23/0.02 (30 °C)	6.48	7.1	0.0228 ± 0.0007	7.91 ± 0.09	3.1 ± 0.3
#3:Control 100/0.23/0.02 (30 °C)	10	7.1	0.0243 ± 0.0009	8.14 ± 0.07	3.5 ± 0.2
#4:100/5/0.5 (30 °C)	10	7.2	0.0539 ± 0.0009	15.1 ± 0.2	8.4 ± 0.5
#5: 100/10/0.5 (23 °C)	10	7.1	0.2443 ± 0.0002	21.6 ± 0.5	11.2 ± 0.6
#6: 100/10/0.5 (30 °C)	10	7.1	0.9134 ± 0.0008	24.2 ± 0.6	12.3 ± 0.5
#7: 100/10/1 (23 °C)	10	7.1	0.8435 ± 0.0007	25.1 ± 0.7	13.2 ± 0.6
#8: 100/10/1 (30 °C)	10	7.1	1.103 ± 0.003	25.9 ± 0.9	13.9 ± 0.6
#9: 100/10/0.5 (23 °C)	13.5	7.1	0.4413 ± 0.0007	22.9 ± 0.8	13.3 ± 0.7
#10: 100/10/0.5 (30 °C)	13.5	7.1	1. 134 ± 0.004	26.2 ± 0.5	15.5 ± 0.6
#11: 100/10/1 (23 °C)	13.5	7.1	1.315 ± 0.003	28.2 ± 0.8	17.5 ± 0.7
#12:100/10/1 (30 °C)	13.5	7.1	1.721 ± 0.007	22.7 ± 0.6	19.9 ± 0.6
#13: D1D2 100/10/0.5 (30 °C)	13.5	7.1	11.341 ± 0.008	46.9 ± 0.8	28.9 ± 0.7
#14: D1D2 100/10/1 (30 °C)	13.5	7.1	14.94 ± 0.01	53.5 ± 0.9	32.1 ± 0.7
#15: D5D1 100/10/0.5(30 °C)	13.5	7.1	10.902 ± 0.009	41.1 ± 0.7	22.8 ± 0.5
#16: D5D1 100/10/1 (30 °C)	13.5	7.1	14.13 ± 0.001	50.8 ± 0.9	29.2 ± 0.8

The statistical analysis using one-way ANOVA with a variance using the 95 % confidence level (p > 0.05) showed that the total biomass and the TPH removal were significantly higher in the biopiles bioaugmented with D1D2 or D5D1 at C/N/P ratios of 100/10/0.5 and 100/10/1 at 30 °C, than all the biostimulated piles. The C/N/P ratio of 100/10/1 was significantly more suitable both for growth and TP removal. D1D2 was also significantly more appropriate than D5D1. Interestingly, both strains D1D2 and D5D1 significantly improved the removal of the oil range organics (*n-*C10- *n-*C35) which include the most difficult hydrocarbons exceeding *n-*C25. The relationship between the growth in term of cfu and TPH removal is an indicator of the adaptation of the bacteria to the available substrate for growth. However, since many bioconversions can also occur and lead to generation of energy to the cells without net removal of the substrate, it is difficult to attribute the growth only to degradation of the substrate.

## 4 Conclusions

The results indicated that hydrocarbon-degrading bacteria can adapt to soil contaminated with highly weathered oil, particularly in the harsh Arabian Gulf environment. Eight and 16 bacterial species were isolated by enriching cultures from soil collected in the polluted AlZubara and Dukhan areas, respectively. The bacteria were identified using the 16S rDNA (*i.e.*, by Ribotyping). *Bacillus* and *Virgibacillus* bacteria were found to be dominant as highly tolerant to 10 % diesel (75 d/L hydrocarbons) in the AlZubara soil, and *Bacillus* and *Pseudomonas* were found to be dominant in the Dukhan soil. The main objective of such isolation procedure by enrichment cultures was to evaluate the potential of the indigenous bacteria to grow and remove the weathered hydrocarbons from soils characterized by harsh conditions for long time. The isolated strains do represent only a fraction of all the indigenous population. The high toxicity pressure of the isolation program lead to the enrichment of the cultures with strains having a potential of application *in-situ* or *ex-situ* if properly bioaugmented in the soil from where they are originated and adapted. Isolated bacterial species had strong activities for all types of TPH, but *Bacillus licheniformis* from the AlZubara soil and *Pseudomonas aeruginosa* from the Dukhan soil had particularly strong potentials for degrading oil, indicated by the increases in the *n-*C17/pristane and *n-*C18/phytane ratios in cultures of these species. Self-purification by endogenous bacteria was found to be possible after the C/N/P ratios for the soil had been adjusted to 100/10/1. Self-purification removed 30 % of the TPH-DRO and 20 % of the TPH-ORO. More interestingly, *Bacillus licheniformis* and *Pseudomonas aeruginosa* removed >50 % of the TPH-DRO and 30 % of the TPH-ORO in 90 d. Interestingly, these results show that the introduced bacteria cooperated with the rest of the endogenous bacteria through co-metabolic activities or commensalism [39]. They did not exhibit inhibition or produce intermediates to other active bacterial strains of importance to the removal of TPH. These findings are important because they indicate that *in situ* bioremediation of sites polluted with strongly weathered hydrocarbons is possible, if highly adapted endogenous bacteria which are isolated though an appropriate isolation and screening program based on high toxicity pressure, are bioaugmented. A similar conclusion, that any spillage problem should be considered separately, and a sustainable strategy based on suitable technology should be developed, was drawn by Ivshina et al. [42].

## Author statement

**Nasser AlKaabi**: Designed the research, performed the experiments, analyzed the data, and drafted the manuscript.

**Nabil Zouari:** Senior Author, supervisor of PhD Thesis of Nasser AlKaabi. Designed the research, analyzed the data, provided equipment and infrastructure, wrote/edited the manuscript.

**Mohammad AlGhouti:** Co-supervisor of PhD Thesis of Nasser AlKaabi. Designed the research, analyzed the data, provided equipment and infrastructure, contributed in writing/editing of the manuscript.

**Samir Jaoua:** Designed the experiments of identification of the bacteria, analyzed the corresponding data, provided equipment and infrastructure, contributed in writing/editing of the manuscript

## Funding

The publication of this article was funded by the Qatar National Library.

## Declaration of Competing Interest

The authors report no declarations of interest.
